# Editorial: Emerging Strategies in Infection Control and Antimicrobial Therapy

**DOI:** 10.3390/medicina62091707

**Published:** 2026-09-06

**Authors:** Doina Azoicăi, Doina Carmen Manciuc

**Affiliations:** 1 Department of Preventive Medicine and Interdisciplinarity, “Grigore T. Popa” University of Medicine and Pharmacy, 700115 Iasi, Romania; 2 Department of Infectious Diseases, “Grigore T. Popa” University of Medicine and Pharmacy, 700115 Iasi, Romania; dmanciuc@yahoo.com

The World Health Organization has repeatedly issued warnings regarding the danger of an escalation in infectious pathology, whether through the appearance of new diseases termed emerging, or through re-emerging diseases resulting from increased morbidity in conditions previously considered controllable and controlled [1,2,3].

Since the seventh decade of the twentieth century, more than 40 emerging infectious diseases have been recorded, including SARS, Ebola, and influenza caused by the AH1N1 and AH1N5 viruses. Taking the year 1918 as a temporal reference point (the devastating pandemic produced by the AH1N1 influenza virus), it is estimated that over the course of approximately 100 years the risks of the emergence and evolution of major epidemiological events—such as extensive epidemics and even pandemics—have intensified. Advanced medical knowledge, diagnostic technologies employed for the purpose of establishing etiology, as well as an unprecedented circulation of populations (resulting from travel, migration, and armed conflicts, all considerably more frequent and occurring over much greater distances than in previous eras) have had the cumulative effect of increasing the capacity for the spread of transmissible infectious diseases. Toward the end of 2019, these concerns were objectively confirmed with the emergence into circulation of SARS-CoV-2, the causative agent of the pandemic that resulted in numerous losses of human life as well as certain paradigm shifts in the evolution of human society.

The burden of diseases generated by the phenomenon of emergence or re-emergence is considerable, both for the individual and for the community as a whole. The long-term consequences are substantial, given that the factors underlying these phenomena are social, economic, environmental, and ecological in nature. At the same time, climate change, together with the multiple elements comprised within the One Health concept, will have a considerable impact on the health of future generations, irrespective of age group, gender, ethnicity, tradition, or social affiliation.

Understanding the mechanisms underlying the emergence and transmission of emerging and re-emerging infectious diseases, through the reconsideration of theories of causality, constitutes a preoccupation of modern medicine, insofar as optimal solutions are required in order to anticipate epidemiological, social, and economic phenomena—potentially of global scale—and, above all, to optimize rapid response in situations of alert. In general, within the biological world, what today appears to be a certainty may, in the near future, prove to be only a partial truth requiring reconsideration in order to ensure the survival and progress of the human species.

Proceeding from an understanding of the notion of emergence, we recognize that humanity may be permanently confronted with such situations, which must be identified and effectively managed.

The “theory of epidemiological transition,” proposed in 1971 by Abdel Omran [4], begins with an initial stage termed “the age of pestilence and famine” and describes successive stages based on the involvement of microorganisms in the production of disease. Beginning with the seventeenth and eighteenth centuries, medical knowledge underwent considerable progress as a result of the understanding of the role of pathogenic agents as a cause of illness, and the contribution of microbiologists was remarkable in opening new perspectives for experimental medicine. Innovative solutions were gradually identified through discoveries in the field of sulfonamide therapy and the first antibiotics, subsequently complemented by preventive measures based on the use of sera and the earliest vaccines. The stage of “receding pandemics” brought about a new outlook regarding the increase in human life expectancy and the decline of major epidemiological events. Closer to the present day, other threats have been identified, arising from the increased risks produced by “modern” behaviors and lifestyles. Thus emerge the challenges of the stage of “degenerative diseases,” in which mortality due to cardiovascular disease, cancer, violence, accidents, and drug use, among other factors, raises new barriers with respect to the health status and quality of life of contemporary individuals. In 1955, the optimistic theories of epidemiologists held that an important shift was being recorded in the history of disease, characterized by a declining interest in the infectious causes of illness, as a result of the limitation of the consequences of pathogen activity, kept under control through preventive and therapeutic measures that had produced remarkable results (vaccination against smallpox, poliomyelitis, and diphtheria, and treatment with antibiotics).

Surprisingly, however, precisely as the notion of a transition from health strategies addressing infectious disease risk toward those addressing non-communicable disease risk was being increasingly promoted, a renewed prominence was observed with respect to threats arising from the aggressiveness of newly circulating microorganisms (emergence) or from the manifestation of diseases previously believed to be under control (re-emergence). Thus, approximately a century after the eradication of smallpox, we are increasingly confronted with new measles epidemics, the appearance of diphtheria cases, and continued setbacks in poliomyelitis eradication strategies as a result of the ongoing circulation of wild-type poliovirus type 1.

Beyond the threats posed by infectious pathology manifesting within the community, Healthcare-Associated Infections (HAIs) constitute a major phenomenon of emergence and re-emergence, owing to antibiotic resistance, the frequency of invasive procedures, and patient vulnerability. Their clinical and social impact is significant, on account of increased costs resulting from prolonged hospitalization, as well as the elevated vital risk arising from severe complications or the markedly increased mortality rate among hospitalized patients.

Antibiotic resistance constitutes a continuous evolutionary process, representing a natural adaptation through the selection of increasingly aggressive bacterial strains over time. When an antibiotic is administered, susceptible bacteria are eliminated, whereas those exhibiting minor favorable genetic mutations survive. The amplification of resistance is expressed through the rapid multiplication of surviving bacteria, which transmit the specific resistance trait to subsequent generations. Furthermore, through genetic transfer, bacteria may exchange resistance genes among themselves—even across different species—thereby accelerating the spread of the phenomenon. The principal causes are multiple and include: the excessive or inappropriate use of antibiotics, the discontinuation of treatment prior to the duration recommended by the physician (thereby permitting the selection of the most resistant bacterial strains), and the excessive use of antibiotics within the veterinary sector [5].

The principal conclusions indicate that the emergence of infectious diseases and antimicrobial resistance (AMR) constitute a major global threat, accelerated by the misuse of medications, climate change, and human mobility—circumstances that necessitate the urgent adoption of the integrated “One Health” concept [6].

Several papers already published in this Special Issue illustrate these challenges with concrete clinical data. Among emergency department patients with sepsis, Gram-positive bacteremia carried a four-fold higher odds of inappropriate empirical antibiotic therapy than Gram-negative bacteremia, and this diagnostic-treatment gap—rather than the pathogen itself—appeared to drive much of the excess mortality observed [7]. A retrospective study from a Romanian emergency hospital found that Watch-group antibiotics predominated over Access-group agents from 2023 onward, falling short of WHO-recommended targets, with Clostridioides difficile and catheter-associated urinary tract infections as the most frequent healthcare-associated infections identified [8]. A related analysis from a tertiary cardiovascular center in Iași reported healthcare-associated infective endocarditis in over 10% of cases, with central venous catheter use nearly quadrupling the risk [9]. In surgical wards, in-hospital mortality was similar between SARS-CoV-2 infection (17.6%) and Clostridioides difficile infection (16.4%), underscoring that CDI remains as clinically significant as pandemic-era viral infection in this setting [10]. The 2024 measles epidemic in Romania, the largest in the EU that year, showed markedly more liver involvement in adults than in children (63.1% vs. 34.2% with elevated transaminases), pointing to a shifting clinical picture as outbreaks increasingly affect unvaccinated adults [11]. Separate work identified renal dose-adjustment errors in nearly 16% of antimicrobial prescriptions in a Greek tertiary hospital, most often involving colistin and cephalosporins [12]; showed that a decline in eosinophil counts at 72 h, rather than at admission, independently predicted mortality in sepsis [13]; and found that metagenomic next-generation sequencing changed antibiotic management in nearly 40% of pediatric cases tested, mostly among immunosuppressed patients [14]. A decade-long surveillance study from Taiwan documented a decline in MRSA infections alongside a rise in carbapenem-resistant Klebsiella pneumoniae following the COVID-19 pandemic, illustrating how resistance patterns can shift even as overall infection control improves [15]. Finally, a narrative review examined which inflammatory, hematological, fecal, renal, and immune biomarkers can meaningfully predict the severity of Clostridioides difficile infection, concluding that markers such as interleukins and presepsin may complement—but not yet replace—established clinical criteria [16].

Solutions aimed at limiting these phenomena must be implemented as rapidly and concretely as possible, through the judicious use of antibiotics, strict control of the circulation of microorganisms within both human and extra-human populations, and the application of preventive measures to reduce risk potential over the coming decades.

## Data Availability

No new data were created or analyzed in this study. Data sharing is not applicable to this article.

## References

[1] Scientific Advisory Group on the Origins of Novel Pathogens (SAGO) (2025). WHO Global Framework to Define and Guide Studies into the Origins of Emerging and Re-Emerging Pathogens with Epidemic and Pandemic Potential.

[2] Wang S., Li W., Wang Z., Yang W., Li E., Xia X., Yan F., Chiu S. (2024). Emerging and reemerging infectious diseases: Global trends and new strategies for their prevention and control. Signal Transduct. Target. Ther..

[3] De Gaetano S., Ponzo E., Midiri A., Mancuso G., Filippone D., Infortuna G., Zummo S., Biondo C. (2025). Global trends and action items for the prevention and control of emerging and re-emerging infectious diseases. Hygiene.

[4] Omran A.R. (1971). The epidemiologic transition: A theory of the epidemiology of population change. Milbank Meml. Fund Q..

[5] World Health Organization (2025). Global Antibiotic Resistance Surveillance Report 2025: WHO Global Antimicrobial Resistance and Use Surveillance System (GLASS)—Summary.

[6] ECDC European Centre for Disease Prevention and Control (2021). The European Union One Health 2021 Zoonoses Report.

[7] Kim T., Yoo J., Lee S., Jung W. (2026). Clinical Characteristics, Empirical Antibiotic Appropriateness, and Outcomes of Gram-Positive Versus Gram-Negative Bacteremia in Emergency Department Patients with Sepsis. Medicina.

[8] Matei M.-C., Chirica V.-A., Ifrim M., Morariu C., Spaiuc D., Manole A., Moscalu M. (2026). Antibiotic Consumption and Healthcare-Associated Infection Surveillance in a Multi-Unit Emergency Hospital in Romania: A Retrospective Observational Study. Medicina.

[9] Matei A., Tinică G., Bacușcă A., Enache M., Țăruș A., Luca M.C., Juganariu G., Azoicăi D. (2026). Changing Epidemiology, Healthcare-Associated Infections, and Outcomes in Infective Endocarditis: A Five-Year Retrospective Study from a Tertiary Cardiovascular Center. Medicina.

[10] Sandu A.M., Vrancianu C.O., Necula M., Cristian R.-E., Păunescu A., Diaconescu D., Tantu M.M. (2026). Clinical Outcomes and Risk Factors of Healthcare-Associated Infections in Surgical Wards: A Retrospective Cohort Study. Medicina.

[11] Vâță A., Miftode I.-L., Grigoriu M.G., Mihuta I., Onofrei I.M., Oancea A.F., Luca M.C., Miftode E.G. (2026). Distinct Clinical and Laboratory Features of Measles in Adults and Children During the 2024 Epidemic: A Retrospective Study from a Romanian Tertiary Infectious Diseases Center. Medicina.

[12] Ioannou P., Papazachariou A., Karakonstantis S., Kofteridis D. (2026). Inappropriate Antimicrobial Dosing in Regard to Renal Function in a Tertiary Hospital in Greece—A Single-Center Point Prevalence Study. Medicina.

[13] Mușat F., Andronic O., Bolocan A., Palcău C.-A., Dobre A.M., Ion D., Păduraru D.N. (2026). Static and Dynamic Eosinophil Measures and In-Hospital Mortality in Patients with Sepsis: A Retrospective Cohort Study. Medicina.

[14] Gonzalez A., Argotsinger J., Oram R.J., Miller J.L. (2026). Impact of Metagenomic Next-Generation Sequencing on Antibiotic Management in Pediatric Patients. Medicina.

[15] Wang C.-Y., Chen Y.-H., Hsiao C.-C., Cheng C.-G., Cheng C.-A. (2025). The Change in Healthcare-Associated Infections in Intensive Care Units Associated with the Coronavirus Disease 2019 in Taiwan. Medicina.

[16] Balas-Maftei B., Florea C.-E., Abudanii L., Stoian I.A., Costin C.A., Grigoriu M., Irimie-Baluta E., Sandu O.-M., Rotaru A., Manciuc C. (2026). Biomarkers for Early Severity Prediction in Clostridioides difficile Infection: Current Evidence, Clinical Utility, and Future Directions. Medicina.

